# *AoB PLANTS*: origins and features

**DOI:** 10.1093/aobpla/plp002

**Published:** 2009-09-18

**Authors:** Michael B. Jackson

**Affiliations:** School of Biological Sciences, University of Bristol, Woodland Road, Bristol BS8 1UG, UK

## Abstract

**Introduction:**

*AoB PLANTS* is a peer reviewed, Open Access (OA) journal owned and run by plant biologists and published by Oxford University Press. The journal publishes research papers, reviews and opinion papers on all aspects of land based plant biology. They are made available rapidly online and can be accessed without the need for subscriptions or payment.

**Background:**

Several difficulties in conventional publishing of peer-reviewed manuscripts encouraged *AoB PLANTS* to adopt OA. Open Access helps sidestep library budgets which are struggling to purchase the increasing numbers of journals. Open Access makes research freely available both to the academic community and beyond while publishing online only eliminates the need to reject good manuscripts simply to control the size of the printed journal. Finally, the journal chose to go OA to ensure the widest possible international readership for the growing amount of high-quality plant science research being carried out worldwide in response to problems such as climate change and food shortages. Responses to a wide-ranging online questionnaire indicated strong international support for a journal such as *AoB PLANTS*.

**Principal features:**

*AoB PLANTS* strives for fair and rapid peer review followed by fast publication of accepted papers. For an initial period, there will be no OA fees, and fees will remain modest once introduced. *AoB PLANTS* adopts double-blind peer review using published criteria for acceptability as a basis for decision-making. Accepted papers are published shortly after acceptance together with referees' analyses using Stanford University Libraries High Wire Press H2O platform. Authors retain ownership of the copyright in their papers.

## Abstract (precis)

The reasons that lie behind starting AoB PLANTS and the ambitions and principal features of the new journal are explained in detail by the Chief Editor.

## Introduction

*AoB PLANTS* is a so-called ‘gold path’ Open Access (OA) journal (Jeffery 2006) dealing with most aspects of plant biology. It is published by Oxford University Press for the Annals of Botany Company, a long-standing not-for-profit organization that supports plant biology internationally (Website 1). The Journal publishes all its accepted papers online within 2–3 d of acceptance and makes them accessible without charge. A broad definition of OA is given by the 2002 Budapest Open Access Initiative (Website 2).

*AoB PLANTS* extends the Annals of Botany Company's publishing activities by complementing its long-standing flagship journal *Annals of Botany*. The initial motivation for *AoB PLANTS* was a sustained and sizeable increase in submissions to *Annals of Botany*, resulting in the rejection of many valuable and important papers which the journal simply could not accommodate. To cater for the general increase in high-quality articles, *AoB PLANTS* was designed as an online-only journal, not restricted by page extent limitations, and which could use the potential of OA publishing to make its articles available quickly to the widest possible readership. Responses to an online questionnaire sent to several thousand plant biologists confirmed that a journal like *AoB PLANTS* would be welcome worldwide.

Although many of the familiar subscription-based journals serve plant biology very well, some uncertainties and difficulties concerning their potential to satisfy future needs and expectations also hastened the founding the *AoB PLANTS*. Several of these are discussed below.

## Some difficulties posed by the conventional publishing model

### Journal pricing and limitations to reader access

**Fig. 1:**
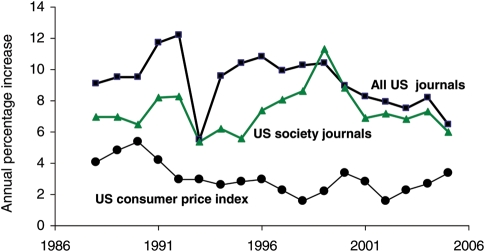
**Average annual increases in prices charged for US journals compared to the annual increase in the US general consumer price index.** Prices are shown for approximately 140 non-profit, association and society STM journals and for a large sample of U.S. periodical titles selected by the American Library Association that include for profit and not for profit publishers. Graph based on tabular data in Kean (2007).

Research budgets have increased year-on-year by approximately 2.5%, and this has resulted in more and more published articles. As journals have increased in size their prices have also increased – many far in excess of the general retail price inflation (Fig. 1). Library budgets have not kept pace with research budgets, and in many cases have been cut, so that librarians have been unable to keep pace with journal prices and the new subscriptions that they wish to take. Reducing subscriptions not only denies access to more academics and researchers but also reduces income for publishers leading to further increases in subscription prices to compensate. A vicious circle is the result, which is bad for science. Open Access journals such as *AoB PLANTS* can help to slow these unwelcome trends by replacing library subscriptions with modest fees from authors or their supporting institutions. If money comes from research grants then Open Access becomes a more sustainable model in an environment of increasing research and publication. This antidote to mounting journal subscription costs for libraries carries the enormous bonus of giving readers within and outside the university/research institution system unrestricted access to the most up-to-date information that is available on the internet and removes many copyright restrictions.

### Increasing science, increasing rejections

**Fig. 2:**
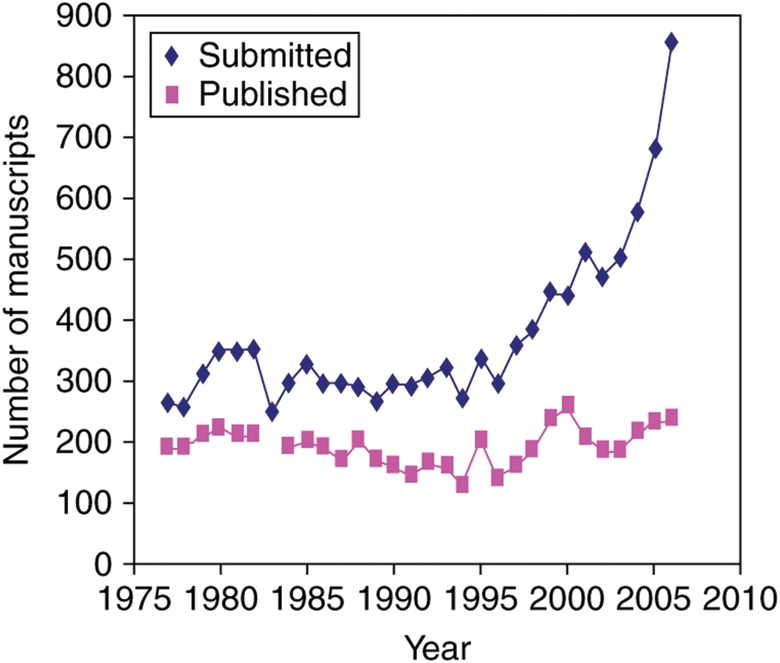
**Numbers of papers submitted to *Annals of Botany* between 1977 and 2006 and the number of papers actually published.** Graph appeared previously in Jackson (2008).

There is an inescapable link between the number of papers published and the costs of production, especially for journals that publish print issues. This deters conventional journals from expanding in proportion to the growth in world science and the number of good manuscripts looking for a home. The increasing gap between numbers of submissions and papers published is exemplified by performance data from *Annals of Botany* (Fig. 2). The outcome over recent years has been an increase in the number of frustrated authors of good papers because popular journals such as this are forced to find ever more refined reasons not to publish manuscripts sent to them. By combining an online-only and OA model, *AoB PLANTS* will largely circumvent the size-cost constraint and offer a genuinely supportive and transparent publishing service that takes the quality, utility and clarity of the science into account unclouded by worries of excessive growth.

## Institutional support for OA publishing

While the majority of authors are funded to carry out research, they are also under increasing pressure to publish their results in ways that make them widely accessible at the earliest opportunity in order to further scientific understanding and benefit society more directly. Open Access publishing facilitates these goals by making articles freely available without charge as soon as they are published. The instant accessibility afforded by OA has been shown to increase PDF downloads in the first 6 months after publication (Davis *et al*, 2008). Thus, readers are more likely to make use of an article if it is published in OA—surely the desired outcome for both authors and their sponsors. In the longer term, citation levels may also benefit from OA (Lawrence, 2001; Hajjem *et al.* 2005; Harnad *et al.* 2008). Not surprisingly therefore, OA publishing enjoys the support of numerous major national and international funding organizations. These include:

- The Wellcome Trust (Website 3).
- Centre National de la Recherche Scientifique (CNRS) (Website 4).
- UK research councils such as the Biotechnology and Biological Sciences Research Council (Website 5).
- US National Institutes of Health (Website 6).

The UK Joint Information Systems Committee (JISC) (Website 7) has recently published an independent cost-benefit analysis of alternative means for scholarly publishing. It concludes that future expansion of OA publishing offers enhanced accessibility and efficiency and an overall economic benefit to the community compared with conventional subscription-based publishing.

These endorsements by major funders of research and by national advisory organisations are indicative of a move towards OA as the preferred publishing option. They rest firmly on principled considerations of august national and international organisations such as the United Nations that see OA as upholding a right of everyone to access publicly-funded information. This is in line with the UN Universal Declaration of Human Rights. Public declarations of support for the OA system and definitions of what constitutes OA include:

- The 2001 Budapest Open Access Initiative (Website 2).
- The 2003 Declaration by the UN-sponsored Geneva World Summit on the Information Society (Website 8).
- The 2003 Berlin Declaration on OA to Knowledge in the Sciences and Humanities (Website 9).
- The 2003 Bethesda Statement on OA Publishing (Website 10).
- The 2003 International Federation of Library Associations and Institutions Statement (Website 11).
- Open Access at the Max Planck Society. Max Planck Society (Website 12).

## Defining features of *AoB PLANTS*

One could argue over the need for new OA plant science journal since there are already one or two other such journals and also a plethora of more conventional titles. But what was missing until now is a genuinely broad spectrum OA journal owned and managed by plant scientists for plant scientists that is not driven primarily by commercial ambition. All members of the *AoB PLANTS* board of management (Website 2) and of its international Advisory Board (Website 13) are highly distinguished plant scientists. Similarly, the Editorial Board (Website 13) comprises prominent plant scientists from many countries and each are invited personally by the Chief Editor. I believe these features, coupled to the long-term commitment of an internationally renowned first**-**division publisher (Oxford University Press), is a winning combination that will attract the confidence of plant biologists worldwide.

All involved with *AoB PLANTS* intend the new Journal to be a long**-**term success. The journal's management will take a flexible and adaptive approach, ensuring that the Journal will evolve to satisfy new needs as they arise, exploit new opportunities and deliver content as effectively as possible to the widest possible virtual community. Many other OA journals are highly specialized and cater for narrow subject areas. *AoB PLANTS* has no such content restrictions thereby widening its access and helping research to reach the entire plant science community. The incorporation of short summaries of each paper, the use of structured abstracts of generous length, and the insistence that Introductions to each paper are aimed at non-specialists will appeal to the widest possible range of readers and give maximum exposure to every paper published in *AoB PLANTS*. A summary of the Journal's main features is given in Table 1.

**Table 1:** Summary of the principal features of *AoB PLANTS*.

Feature	Description
Scope	*AoB PLANTS* publishes original research and review papers on land-based plant biology. It welcomes interdisciplinary and specialised topics on theoretical, experimental, pure or applied aspects.
Purpose	*AoB PLANTS* provides an affordable OA route for the rapid publication of peer reviewed manuscripts assessed against clearly defined criteria of acceptability.
Costs to authors	Authors currently pay nothing to publish in *AoB PLANTS*. Later, charges will increase slowly but will remain competitive. Submitting is cost-free.
Excellence of the science	Research papers should be hypothesis driven, based on well-executed and appropriate methods, extend current knowledge and make clear, soundly-argued conclusions.
Fast, high-quality publication	Content is available on-line without charge within days of acceptance. High Wire Press platform provides many helpful features including focussed alerting, ETOCs, extensive links to cited papers and authors, E-Letters comments system.
Open Access	Authors and readers enjoy unrestricted toll-free use, distribution and reproduction of content provided the original source in *AoB PLANTS* is quoted.
Fast, fair peer review	Double blind refereeing. Submissions assessed against clearly defined minimum acceptance criteria emphasising technical excellence, reproducibility, and clarity. Highly international Editorial Board. Referee commentary for accepted papers.
Author services	On-line submission, fast and fair evaluation process, figures re-drawn, author correction of proofs, control of copyright and deposition rights.
Features of the papers	Introductions with non-specialist content. 300-word structured abstracts. PDF versions with spacious colour-enhanced layout. Papers accompanied by 30-word illustrated summaries.
Distribution	Papers searchable in Google will be deposited on PubMed Central and other providers such as *Directory* of *Open Access Journals* (Website 14) and Twitter.

Although author fees will always be a very small proportion of the total cost of the research, *AoB PLANTS* recognizes that funds are often hard-won. Accordingly, it will make every effort to minimise the costs. This policy is in keeping with the Journal's not-for-profit ethos. For an initial period, authors will pay no fees, all costs being paid in full by the Journal's owners. In order to remain sustainable, authors will start to be charged at a later stage. However, the cost will increase slowly in several stages but remain highly competitive.

## Conclusions and forward look

*AoB PLANTS* is designed to support the growing number of authors and funding institutions wishing to publish their articles in an OA journal, arguably the most effective and accessible publishing medium. Readers will benefit from cost-free access to the latest peer-reviewed papers while authors will see their articles appearing quickly and attracting universal reader attention while the information is still fresh. This is vital as plant science expands to meet the challenges of climate change, pollution and pressures from human population growth. Authors and readers will also have opportunity to reuse, extract and display content for scholarly purposes without the troublesome need for permissions as long as the original source in *AoB PLANTS* is stated and the authors given full credit. The OA approach will also make it more straightforward for *AoB PLANTS* to accommodate growth of plant science research worldwide and the attendant increase in manuscript numbers. *AoB PLANTS'* not-for-profit constitution also means it can offer authors a no-cost or low-cost high quality alternative to commercial profit-driven competitors.
